# Strabismus Surgery for Esotropia, Down Syndrome and Developmental Delay; Is an Altered Surgical Dose Required? A Literature Review

**DOI:** 10.22599/bioj.140

**Published:** 2020-02-05

**Authors:** Alia Harrison, Louise Allen, Anna O’Connor

**Affiliations:** 1 University of Liverpool, GB

**Keywords:** Strabismus, Esotropia, Surgery, Down Syndrome, Developmental Delay

## Abstract

**Background and Purpose:**

There is a high rate of strabismus, in particular esotropia, in children with Down syndrome or developmental delay, which frequently requires surgical correction. A paper in 1994 advocated that the surgical dose be adjusted due to an altered response in these children. The aim of this literature review is to evaluate the available evidence to establish whether an altered surgical approach is required in either population.

**Methods:**

A literature review was conducted using PubMed and Web of Knowledge. Only English language papers were eligible for inclusion. The papers were collated in chronological order for analysis, and their references searched for further relevant papers. Forward citation searches were also undertaken.

**Results:**

A 2 × 2 comparison is made between publications on Down syndrome (in isolation) and developmental delay populations (including Down syndrome) with adjusted versus non-adjusted surgery. Published surgical success rates on esotropia from unaltered surgical doses range from 62.0%–85.7% (four papers) in the Down syndrome cohort, with none of the adjusted surgeries having a successful outcome. Surgical success rates from adjusted surgical doses in developmental delay cohort range from 37.5%–86.0% (seven papers), with one unadjusted surgical success rate of 76.0%. The results across the studies are summarised in a table and discussed.

**Conclusions:**

An exaggerated surgical effect in individuals with developmental delay has been reported, and this population may benefit from a reduced surgical dose. Published research does not support giving a reduced surgical dose in individuals with Down syndrome, but more research needs to be done to make a definitive conclusion.

## Introduction

Strabismus is a common ocular manifestation of Down syndrome; incidence figures range between 19% and 42% (Haugen & Hovding 2001; Cregg et al. 2003; Yurdakul et al. 2006; Ljubic et al. 2011; Ljubic et al. 2015), compared to the 2%–5% incidence in the general population (Robaei et al. 2006; Friedman et al. 2009). Of those with strabismus, 71%–100% (Haugen & Hovding 2001; Cregg et al. 2003; Yurdakul et al. 2006; Ljubic et al. 2011; Ljubic et al. 2015) are reported to have esotropia, although the specific types of esotropia are not stated. Similarly, it has also been reported that incidence of strabismus is increased in children with developmental delay, with figures ranging from 17.4–40.0% (Bankes 1974; Nielsen et al. 2007; Das et al. 2010). Again, a trend towards esotropia has been reported, although in the developmental delay population figures for esotropia range between 14.9% and 76.8% of strabismus cases, suggesting more variability in this population.

While the increased prevalence of strabismus is known, the aetiology of the strabismus in both populations is not fully understood. However, within the Down syndrome population it is well documented that in combination with the increased rates of strabismus, the incidence of hypermetropia is also increased. Although the increased rates of hypermetropia may be a significant factor resulting in the increased rates of strabismus, Cregg et al. (2003) reported a lack of a statistical association between strabismus and hypermetropia in their longitudinal study (p = 0.539, n = 55, representing approximately just 0.14% of the UK population of individuals with Down syndrome). Given the lack of association, it was theorised that an aspect of the aetiology may be as a result of hypotonic extraocular muscles, congruous in the context of the generalised hypotonia of individuals with Down syndrome (Corrêa et al. 2011). Considering that the manifest strabismus may not be corrected with spectacle lenses, individuals may wish to consider strabismus surgery to improve their ocular alignment.

This paper aims to review existing reports regarding techniques and amounts of surgery, in order to establish whether surgical doses should be adapted for individuals with Down syndrome. The available literature has often included individuals with Down syndrome in cohorts studied under the term of ‘developmental delay’. Developmental delay is an umbrella term for children experiencing delay in two or more areas of development categories: fine/gross motor skills, speech and language development, cognition, social/personal, and activities of daily living (Majnemer & Shevell 1995; Shevell et al. 2003; Mithyantha et al. 2017). Developmental delay may occur alone, or alongside another condition. As the term covers children with a wide range of developmental abilities it can be open to interpretation; for example, individual states in the US have their own definition of developmental delay, and differing age ranges to which the diagnosis applies (Hadadian & Koch 2013). Due to this scope in definition, literature available has often studied cohorts with a variety of conditions, including Down syndrome, under the term ‘developmental delay’. As a result, the scope of this review will also cover individuals described as having developmental delay, whilst focusing on the cohort with Down syndrome. In addition, this review and the literature covered will primarily consider the surgical effect on esotropia due to the high incidence of esotropia in the populations studied.

## Methods

The starting point for this literature review was the article by Pickering et al (1994). Working from this point, a citation search was performed using Web of Science, returning 26 citations. Articles considering strabismus surgery in individuals with Down syndrome or developmental delay were selected for inclusion (total n = 7, including the original Pickering et al 1994 article). Articles pertaining to strabismus surgery in individuals with cerebral palsy, prematurity and infantile esotropia, as identified in the title of the article, were excluded due to the effect of these diagnoses on surgical outcomes requiring separate analysis. Articles written in any language other than English (n = 1) were excluded. One review article (Liu & Ranka 2014) was not selected for inclusion to avoid possibly including bias from secondary interpretation; however, the references were used to find further original research articles for inclusion (total for inclusion n = 10).

Searches of PubMed using the terms ‘strabismus’, ‘developmental’, and ‘delay’, as well as ‘strabismus’, ‘down’, and ‘syndrome’ returned 280 and 157 results respectively. Articles relevant to this literature review, as established by their title, but not identified using searches described previously, were selected using the above criteria, with one further article selected for inclusion (total articles included n = 11).

## Results

Table 1 summarises the studies examining surgical effect in populations under the heading developmental delay and in populations with Down Syndrome.

**Table 1:** Summary of Research Studying Participants Under the Terms Developmental Delay and Down Syndrome.

Authors	Study Design	Participants	Age	Strabismus	Surgery	Surgical Adjustment	Outcome (success = within 10^ of orthotropia)

Pickering et al (1994)	Retrospective Review	N = 94 31 DD group 23 known diagnosed condition	Mean 25 months (range 10–80 months)	Esotropia 49^, range 25^–70^ DD group	BMRR	“less surgical correction” performed for “some” children in DD group	69% success at 1 month post op DD group
		63 typically developing controls (TD)	Mean 26 months (range 7–202 months)	50^, range 18^–95^ TD group		No adjustment given to TD group	82% success at 1 month post op TD group
Pickering et al (1995)	Retrospective Review	N = 91 (some previously included in 1994 study) 29 DD group 18 known diagnosed condition	Mean 25 months (range 10–80 months)	Esotropia 49^, range 25^–70^ DD group	BMRR	“less surgery” given to children in DD group after 1987 average 0.54 mm per muscle	DD pre-1987 76% success DD post-1987 86% success (no n number given)
		62 TD controls	Mean 26 months (range 7–202 months)	50^, range 18^–95^ TD group		No adjustment given to TD group	TD pre-1987 82% success TD post 1987 83% success
Habo-Wilner et al (2006)	Retrospective Review	N = 34 16 DD group 8 known diagnosed condition	3.7 ± 2.7 yrs	Esotropia 53 ± 12^ in DD group	BMRR	40^–40^ reduced by 1 mm per muscle 25^–35^ reduced by 0.5 mm per muscle	56% (n = 9) within 10^ of orthotropia. 86% of surgical failures under corrected in DD group
		18 TD controls		37.4 ± 8^ in TD group		No adjustment given to TD group	94% (n = 17) success in TD group
van Rijn et al (2009)	Retrospective Review	N = 104 37 DD group	69.3 ± 41.3 months	Esotropia Angle at 2.5 m 16.6 ± 7.2°	11 single recession 16 BMRR 10 R+R	Study group given “roughly halved” doses for single recessions and BMRR after June 2004	Pre-June 2004 average angle at 2/12 f/up –4.09 ±6.44° (n = 11). Post June 2004 average angle at 2/12 f/up +1.25 ±4.14° (n = 16) (p = 0.028)
		67 TD controls	59.2 ± 31.5 months	Angle at 2.5 m 14.4 ± 7.7°	16 single recession 27 BMRR 24 R+R	No adjustment given to TD group	
Habo-Wilner et al (2012)	Retrospective Review	N = 24 (some previously included in 2006 study) 9 known diagnosed condition 15 non-specific delay	2.8 ± 2.5 yrs, (range 0.8–10 yrs)	Esotropia 49.8^ ± 13.3^	BMRR	Mean dose of 5.1 mm ±0.7 mm per muscle (authors report an average of 0.75 mm less than standard)	38% (n = 9) success. 67% of surgical failures (n = 10) under corrected
Swaminathan (2014)	Retrospective Review	N = 78 25 DD group 10 known diagnosed condition	4.96 ± 3.25 yrs	Esotropia 44.4^ ± 13.25^	BMRR	Greater surgical under correction planned for DD group	60% (n = 15) success, 28% (n = 7) under corrected in DD group
		53 TD controls	5.037 ± 2.205 yrs	39.25^ ± 10.35^		No adjustment given to TD group	74% (n = 39) success, 26% (n = 14) under corrected in TD group
Zehavi-Dorin et al (2016)	Retrospective Review	N = 42 17 known diagnosed condition 25 non-specific delay	Mean 2.9 yrs (range 0.8–10yrs)	Esotropia 44.29^ ± 13.9^ (range 20^–80^)	BMRR	Mean dose of 0.66 m (range 0–1. mm) less than surgical tables	57% (n = 24) success 31% (n = 13) under corrected
Ruttum et al (2004)	Retrospective Review	N = 21 DS	Mean 55 (±35) months	Esotropia	17x BMRR 2x R+R 2x unknown	None–surgical methodology not discussed further	67% (n = 14) within 10^ of orthotropia
Yahalom et al (2010)	Retrospective Review	N = 15 DS (14 outcomes analysed)	Mean 6.2 yrs (range 1.2–24.9 yrs)	14x infantile esotropia	12x BMRR	Standard surgical tables used.	86% (n = 12) within 10^ of orthophoria
				1x constant esotropia with an accommodative element	3x R+R	1x deliberate undercorrection, not included in statistical analysis	
Perez et al (2013)	Retrospective Review	N = 17 DS	Mean 5.9 (±3.8) yrs	10x infantile esotropia 7x acquired esotropia	BMRR	Standard surgical tables used in both groups	76% (n = 13) within 10^ of orthotropia
	Case Control	N = 27 Matched controls	Mean 5.1 (±2.8) yrs	14x acquired esotropia	BMRR		85%(n = 23) within 10^ of orthotropia
Motley et al (2012)	Retrospective Review	N = 16 DS	Median 4.1 (IQR 3.2–6.8) yrs	Esotropia	BMRR	Standard surgical tables used in both groups	No statistical difference in outcome between groups. Success rates not given (P = 0.850)
	Case Control	N = 16 Matched controls	Median 4.6 (IQR 3.5–5.9) yrs				

DS – Down Syndrome. TD – Typically Developing. BMRR – Bilateral Medial Rectus Recessions. R+R – Medial Rectus Recession with Lateral Rectus Resection. ^ – Prism Dioptres.

## Informed Discussion

Pickering et al (1994) were among the first to investigate surgical response in children that had developmental delay. They advocated a reduced surgical dose for children with developmental delay, after retrospectively analysing the surgical outcomes of 31 children with developmental delay and 63 typically developing children. Of the children with developmental delay, 17 were classified as having neurological conditions such as cerebral palsy, hydrocephalus and seizures, six had chromosomal abnormalities, including Down syndrome, whilst the remaining eight patients had non-specific developmental delay. The authors had anecdotally noted a trend towards overcorrection in the developmental delay population. Comparison of outcomes between children with developmental delay and typically developing children undergoing bilateral medial rectus recessions between 1981 and 1991, demonstrated that the children with developmental delay experienced on ‘average’ (presumably mean, but not specified and no standard deviation given) an extra 5.28 prism dioptres in surgical correction towards exotropia. However, while this difference between the two groups was statistically significant at one month postoperatively (p = 0.04), the difference was not significant at one year postoperatively (p = 0.08). For children receiving 4.0 mm recessions of each medial rectus the surgical effect was in fact greater towards exotropia in the typically developing children than in the developmentally delayed children, at both one month and one year postoperatively, although this data point was not individually analysed for statistical significance. While the authors concluded that their findings supported reducing the surgical dose for individuals with developmental delay, this conclusion is not supported by their data.

In a follow up study a year later, Pickering et al (1995) examined how performing reduced amounts of surgery on children with developmental delay impacted on the outcome of their surgery. Success was defined as alignment within 10 prism dioptres of orthophoria. Statistical analysis was not significant when comparing the outcomes between the two groups when they received the same amount of surgery (p = 0.20, total participants across both groups n = 91), and when the surgical dose was altered in the developmental delay group (p = 0.70, n = 29). Despite statistically insignificant results Pickering et al (1995) advocated performing reduced bimedial rectus recessions in children with developmental delay. No specific reduction was suggested; however, the authors reported that they reduced their surgeries by an average of 0.54 mm, with no standard deviation given.

Based on the work by Pickering et al (1994, 1995), some surgeons opted to reduce their bimedial recession in patients with developmental delay. Habot-Wilner et al (2006) used reduced surgical doses, retrospectively reviewing case notes of 34 children: 16 with developmental delay, of whom two had a diagnosis of Down syndrome, and 18 typically developing controls that underwent bimedial rectus recession between 1993 and 2003. Those in the developmental delay group were given a deliberately reduced surgical dose, by a mean of 0.84 mm per muscle (no standard deviation given). This approach resulted in a 56% (n = 9) success rate in the developmental delay group, defined as alignment within 10 prism dioptres of heterotropia. Of the other participants in the developmental delay group 38% (n = 6) of the children were left with an undercorrection of greater than 10 prism dioptres, with 6% (n = 1) being overcorrected with exotropia of greater than 10 prism dioptres. In contrast, in the typically developing group the success rate was 94% (n = 17) with no cases of undercorrection and just one case of overcorrection.

The results of Habot-Wilner et al (2006) demonstrated that a reduction of surgery by an average of 0.84 mm per muscle in the developmentally delayed population resulted in a statistically significant increase in surgical failure (undercorrection of >10 prism dioptres), compared with the typically developing group receiving standard surgery. Despite this statistically significant result, the authors concluded that the ideal surgery for the developmentally delayed population could not be defined, due to the majority of children in both study groups only being followed up for one year. It is also difficult to determine an ideal amount of surgery in the developmentally delayed population from this work as the reductions of 0.84 mm per muscle performed are greater than those suggested by Pickering et al (1995). Therefore, whilst the results of Habot-Wilner et al (2006) do not support reductions of 0.84 mm per muscle, they cannot dismiss the possibility that smaller surgical reductions may lead to improved outcomes in patients with developmental delay.

## Expressing the Surgical Dose

It can be helpful to consider the surgical dose expressed as a percentage correction of the strabismic angle (using surgical tables), rather than in millimetres of muscle adjustment, to understand the impact of alteration of the surgical dose (Swaminathan et al. 2014). Swaminathan (2014) reported on a case control study of 25 children with developmental delay (excluding Down syndrome) and 53 typically developing controls, described as having concomitant esotropia. The authors calculated expected surgical effect for the bimedial rectus recessions performed, and expressed both the amount of surgery performed for the size of the deviation, and the outcome of the surgery for the dose given as a percentage. The difference in surgical dosing between the two groups was statistically significant (p = <0.0001), with surgical dose being reduced in the developmental delay group. Children in the developmental delay group received surgical dose for 72% (±16.08) of the angle of deviation, and typically developing controls received 89% (±10.83). This reduced surgical dose resulted in surgical success rates, within 10 prism dioptres of orthophoria, of 60% (n = 25) and 74% (n = 53) in the developmental delay and control groups respectively. This would suggest that individuals with developmental delay do have an exaggerated response to surgery; deriving greater surgical effect from less percentage correction, and therefore do benefit from reduced surgical dosing to achieve comparable outcomes with typically developing individuals as purported by Pickering et al (1994, 1995). The difference in outcomes between the two groups is however statistically insignificant (p = 0.3) and relates to outcomes at only six weeks post-surgery, a very short follow up period. While studies (Pukrushpan 2009; Wang & Wang 2012) including by PEDIG (2009) have indicated that outcomes at six weeks and six months post op are not significantly different, six months is still a relatively short follow up period, particularly in a paediatric population where six months represents a period of significant growth. Follow up over a 15 year period has shown decreasing surgical success rates over time for children undergoing standard surgery for infantile esotropia (Chatzistefanou et al. 2018), and therefore while the reported surgical outcomes at six weeks may be expected to be comparable at six months, they may not be representative of longer term outcomes, particularly for those children undergoing augmented surgery where natural history of the strabismus is less understood.

## Long Term Follow Up

In order to try and establish the ideal surgical dose, two follow up studies were undertaken at the same centre (Habot-Wilner et al. 2012; Zehavi-Dorin et al. 2016). Habot-Wilner et al (2012) reported on children meeting their inclusion criteria at their centre between 1993–2009. Their previous study reported on the years 1993–2003, and there is therefore some overlap between the two works. In this follow up study the average surgical reduction was 0.75 mm per muscle, range 0–1.5 mm (previously average 0.84 mm reduction per muscle). After one surgery success rates were still poor at 38% (n = 9) with 42% (n = 10) under corrected and 21% (n = 5) overcorrected. Eight of the 15 surgical failures opted for further surgery, with seven children receiving one additional surgery, and one child receiving two additional surgeries. The overall success rate after all additional surgeries was 61% (n = 24). Although this follow up study reported lower surgical success rates with smaller surgical reductions, other authors from the same centre reported conflicting results.

Zehavi-Dorin et al (2016) reviewed surgical outcomes of 42 children with developmental delay, of whom four had Down syndrome and 13 had another specific diagnosis. The children underwent bilateral medial rectus recession, on average reduced by 0.66 mm from the standard dose (range 0–1.5 mm) and were followed up for median 3.67 years (range 8 months to 15 years). This resulted in a surgical success rate of 57% (n = 24), defined as heterotropia of less than 10 prism dioptres, with 31% (n = 13) under corrected and 12% (n = 5) overcorrected, congruent with the 60% success and 12% overcorrection rates reported by Swaminathan et al (2014) in their developmental delay population. These success rates are greater than those reported by Habot-Wilner in their original and follow up studies, where their data had appeared to show lower surgical success rates, with smaller surgical reductions. Zehavi-Dorin et al (2016) also examined the longer-term data available for 16 children who had been followed up for five years, finding that after five years surgical success was 43% (n = 7), with 37.5% (n = 6) undercorrected and 18.5% (n = 3) overcorrected. This could suggest a longer-term trend towards overcorrection in the developmental delay population, and support an argument for initial under correction for longer term success to account for post-operative drift (Park et al. 2009).

## Type of Surgery

Whilst both Pickering et al (1994, 1995) and Habot-Wilner et al (2006) only examined the surgical effect of bimedial rectus recessions in the developmentally delayed population, van Rijn et al (2009) studied both bimedial rectus recessions and unilateral recess/resect procedures. van Rijn et al (2009) reported that whilst there was no significant difference in the outcomes of developmentally delayed children (including four with Down syndrome) compared to typically developing children undergoing unilateral recess/resect procedures (p = 0.918 at 30 cm fixation distance), there was a significant difference between the two groups for those undergoing bimedial rectus recessions (p < 0.001 at 30 cm fixation). The deviations were however measured deviations using a Maddox method. This was a custom-made device chosen by the authors to avoid the possibility of inducing proximal vergence by using prisms, although no published evidence to support their argument has been found. Typically, Maddox methods still require a rod or wing device to be placed in front of the eye, also inducing proximal vergence and therefore negating the authors reasoning. The methodology described measuring deviations fixing both right and left eye and did not report any alternative measurement process. Given that the authors studied children with developmental delay, some of whom were as young as 18 months old, it is difficult to be confident that subjective measurements have given accurate results to analyse. It could however be postulated that as the methodologies between the two groups are the same, this does not impact on the authors’ conclusions, it simply prevents replication.

van Rijn et al (2009) were unable to explain the causation for the difference in outcomes between the surgeries, but theorised that the difference was due to the balance between the tone of the lateral and medial rectus muscles being disturbed by bimedial rectus recessions, but not by unilateral recess/resect procedure. The relevance of this theory for individuals with Down syndrome is difficult to ascertain, given the possibility that the tone of the extra ocular muscles may be altered in individuals with Down syndrome. Individual patients will have different visual development experiences, and therefore limiting the explanation for difference in outcomes only to muscle tone may be artificial. This explanation does not consider the impact of visual acuity, which is also known to be reduced in individuals with Down syndrome (Zahidi et al. 2018), or of the presence or absence of binocular single vision which influence the surgical outcome (Kiziltunc et al. 2016).

## Surgical and Strabismus Measurement Technique

When evaluating the surgical outcomes, the accuracy of the measurements must be considered, both of the surgical measurements and of the measurement of strabismus. Castroviejo callipers used in strabismus surgery mark only to the whole millimetre, and do not have markings for micrometres. It should also be considered that at such small distances, the positioning of the callipers on the sclera, and whether the measurement is taken from the inside or outside edge of the calliper, will have a proportionally greater impact on the accuracy of the measurement than for larger distances. A surgeon could not therefore measure 0.54 mm or 0.84 mm using standard surgical equipment, casting doubt on the validity of the findings and recommendations of Pickering et al (1994) and Habot-Wilner et al (2006).

Strabismus measurement methodology is also pertinent, given that the research explored uses angle of strabismus to define surgical success. Accurate measurement is therefore required in order to reach robust conclusions on surgical success. Several studies discussed herein (Pickering et al. 1994; Pickering et al. 1995; Yahalom et al. 2010) measured deviations using the prism cover test, and the Krimsky method where this was not possible. Krimsky methods and alternate prism cover test (APCT) were examined by Joo et al (Joo et al. 2013), reporting statistically significant (p < 0.001) intraobserver agreement for standard near Krimsky, modified distance Krimsky and APCT methods. When measuring esotropia the Pearson correlation coefficient for near Krimsky and distance APCT was 0.651 (p = 0.003), and 0.695 (p = 0.001) for distance Krimsky and distance APCT. These findings would suggest good intraobserver agreement between the tests, and strong positive correlation between distance Krimsky and distance APCT, with slightly weaker correlation between near Krimsky and distance APCT.

While Krimsky and distance APCT may have strong positive correlation, suggesting good agreement between the two measurement methods, a test must also be repeatable. The testretest variability of the APCT, researched on adults with sixth nerve palsy, and based on 95% confidence intervals has been reported as being 10.2 prism dioptres for distance fixation and 9.2 prism dioptres for near fixation (Holmes et al. 2008). A measurement difference of 5.28 prism dioptres, given as the difference in outcome between the developmentally delayed and typically developing groups in the work by Pickering et al (1995), is easily accounted for by test retest variation, and this difference could therefore be considered clinically insignificant.

## Research Exclusively in the Down Syndrome Population

Whilst the research discussed thus far examines the surgical effect of strabismus surgery on groups of participants with developmental delay, including those with Down syndrome, it is not possible to specifically analyse the outcomes of just the participants with Down syndrome from the published results. Other authors (Motley et al. 2012; Perez et al. 2013; Ruttum et al. 2004; Yahalom et al. 2010), however, studied only participants with Down syndrome. Their findings are summarised in Table 1.

As shown in Table 1, there is some variation in outcomes, and in how the outcomes are measured and reported between studies. Unlike others, Ruttum et al (2004) did not use a control or comparison group, and did not reduce the surgical dose given; the authors instead compared their success rates to those in the published literature. Their success rate of 67% (n = 14) is higher than the success rates of 56% (n = 16) reported by Habot-Wilner et al (2006) and the 60% (n = 25) success rate reported by Swaminathan et al (2014), both of whom used reduced surgical doses. This could suggest reduced surgical doses may result in a poorer success rate, although the research populations have differing characteristics between these studies.

## Participant Demographics

Whilst Ruttum et al (2004) achieved higher success rates than Habot-Wilner (2006) with standard surgical dosing, suggesting that the reduced surgical dose has little impact on outcomes, the demographics of the study groups in terms of diagnosis and surgical dosing are different. Participant demographics are also relevant to Yahalom et al (2010), who described 14 of their patients as having infantile esotropia. Studies exclusively studying infantile esotropia were not included within the scope of this literature review as an individual with Down syndrome may be precluded from a diagnosis of infantile esotropia; since infantile esotropia can be considered to occur in the absence of any other neurological abnormality (Louwagie et al. 2009). This type of strabismus in an individual with Down syndrome may alternatively be diagnosed as a non-accommodative esotropia, as done by Habot-Wilner et al (2006). It could, however, be argued that some neurological impairments may cause infantile esotropia, and that the two diagnoses are not mutually exclusive (Costenbader 1961; von Noorden 1988; Charles & Moore 1992; Simonsz & Kolling 2011), or that infantile esotropia itself is a neurological disorder (Brodsky 2018). Other authors (Ruttum et al. 2004; Motley et al. 2012) however gave no classification of esotropia in their study group, particularly problematic for Ruttum et al (2004) as they compared their results to two studies, one exclusively concerning infantile esotropia, the other one acquired esotropia. It could be argued that this comparison is invalid since the surgical timing and outcomes are different in different types of strabismus (Christiansen et al. 2008).

## Surgical Success in Individuals with Down Syndrome

Yahalom et al. (2010) report the highest surgical success rates of all the Down syndrome population studies identified. One child in their series was given a reduced surgical dose as the surgeon was influenced by literature advocating this approach, however this resulted in an unsuccessful surgery outcome of residual esotropia of greater than 10 prism dioptres. The other participants in this series receiving a standard surgical dose do not appear to have shown an exaggerated surgical response. This finding was replicated by Perez et al (2013), who overcame the difficulties of comparing between studies by using control groups to compare surgical outcomes following bimedial recessions between children with Down syndrome and typically developing children. Both groups received standard surgical doses from the same surgeon. The difference between the success rates in the two groups was not statistically significant (p = 0.46, n = 44). Motley et al (2012) also reported no significant difference between surgeries performed on their two matched groups, with no statistically significant difference in outcomes between the two groups over a 24-month follow-up period (p = 0.8050, n = 32). Information on methodology was however extremely limited, with no details given on measurement of strabismus; an important measure to consider when reviewing outcomes, as discussed previously.

In contrast to the conclusions of van Rijn et al (2009), the findings of Yahalom et al (2010), Motley et al (2012) and Perez et al (2013), would suggest that children with Down syndrome do not have an exaggerated response to bimedial recessions. The success rate reported by Yahalom et al (2010) was 86% (n = 12); very similar to the 85% (n = 23) success rate in typically developing children reported by Perez et al (2013) who did not have a statistically significant difference in outcomes between their two groups (p = 0.46). This would suggest agreement in the findings between the two studies, and broadens support for standard strabismus surgery in individuals with Down syndrome.

## Conclusion

The evidence discussed here has demonstrated that for individuals with Down syndrome there is no clear evidence to support giving a reduced surgical dose, as standard surgery gives outcomes comparable to those of typically developing children receiving standard surgery. Although the cohorts of individuals with Down syndrome in all studies are small, reducing the reliability of individual studies, this finding has been replicated across the studies discussed. While published research is limited to retrospective reviews of esotropia surgery, with the type of esotropia not well classified, and the subclassification not analysed as a variable influencing the success of strabismus surgery, there is no evidence to suggest that the research findings are not applicable to other types of strabismus within the same population. The limitations regarding analysis described above leave the possibility that confounding variables may be influencing results.

Literature studying individuals under the umbrella term of developmental delay would suggest that there may be some populations under this term that could benefit from reduced surgical dosing. The field would benefit from prospective trials involving participants with unifying diagnoses to identify these populations.

## References

[1] Bankes, JLK. 1974 Eye defects of mentally handicapped children. British Medical Journal, 2: 533 DOI: 10.1136/bmj.2.5918.533 4135149 PMC1610946

[2] Brodsky, MC. 2018 Essential infantile esotropia: Potential pathogenetic role of extended subcortical neuroplasticity. DOI: 10.1167/iovs.18-23780 29677359

[3] Charles, SJ and Moore, AT. 1992 Results of early surgery for infantile esotropia in normal and neurologically impaired infants. Eye (London, England), 6(6): 603–606. DOI: 10.1038/eye.1992.130 1289137

[4] Chatzistefanou, KI, Brouzas, D, Droutsas, KD, Koutsandrea, C and Chimonidou, E. 2018 Unilateral recession-resection surgery for infantile esotropia: Survival of motor outcomes and postoperative drifts. Seminars in Ophthalmology, 33: 498–505. DOI: 10.1080/08820538.2017.1312465 28488915

[5] Christiansen, SP, Chandler, DL, Holmes, JM, Arnold, RW, Birch, E, Dagi, LR, et al. 2008 Instability of ocular alignment in childhood esotropia. Ophthalmology, 115: 2266–2274. DOI: 10.1016/j.ophtha.2008.08.011 18973948 PMC2597336

[6] Corrêa, JCF, De Olivera, AR, Oliviera, CS and Correa, FI. 2011 Existence of neurophysiologic changes can assist in understanding the role of hypotonia in motor development of subjects with Down syndrome? Fisioterapia e Pesquisa, 18: 377–381. DOI: 10.1590/S1809-29502011000400014

[7] Costenbader, FD. 1961 Infantile esotropia. Transactions Of The American Ophthalmological Society, 59: 397–429. 13881627 PMC1316420

[8] Cregg, M, Woodhouse, JM, Stewart, RE, Pakeman, VH, Bromham, N, Gunter, HL, et al. 2003 Development of refractive error and strabismus in children with Down syndrome. Investigative Ophthalmology & Visual Science, 1023 DOI: 10.1167/iovs.01-0131 12601024

[9] Das, M, Spowart, K, Crossley, S and Dutton, GN. 2010 Evidence that children with special needs all require visual assessment. Archives of Disease in Childhood, 95: 888 DOI: 10.1136/adc.2009.159053 20647259

[10] Friedman, DS, Repka, MX, Katz, J, Giordano, L, Ibironke, J, Hawse, P, et al. 2009 Prevalence of amblyopia and strabismus in white and African American children aged 6 through 71 Months: The Baltimore pediatric eye disease study. Ophthalmology, 116: 2128–2134.e2. DOI: 10.1016/j.ophtha.2009.04.034 19762084 PMC2783780

[11] Habot-wilner, Z, Spierer, A, Barequet, IS and Wygnanski-Jaffe, T. 2012 Long-term results of esotropia surgery in children with developmental delay. Journal of AAPOS, 16: 32–35. DOI: 10.1016/j.jaapos.2011.10.013 22370662

[12] Hadadian, A and Koch, KR. 2013 Issues in labeling young children with developmental delay: Whose responsibility is It? International Journal of Early Childhood Special Education, 5: 187–199. DOI: 10.20489/intjecse.107932

[13] Haugen, OH and Hovding, G. 2001 Strabismus and binocular function in children with Down syndrome: A population-based, longitudinal study. Acta Ophthalmologica Scandinavica, 79: 133–139. DOI: 10.1034/j.1600-0420.2001.079002133.x 11284750

[14] Holmes, JM, Leske, DA and Hohberger, GG. 2008 Original article: Defining real change in prism-cover test measurements. American Journal of Ophthalmology, 145: 381–385. DOI: 10.1016/j.ajo.2007.09.012 18045567 PMC2386860

[15] Joo, KS, Koo, H and Moon, NJ. 2013 Measurement of strabismic angle using the distance Krimsky test. Korean Journal Of Ophthalmology, 27: 276–281. DOI: 10.3341/kjo.2013.27.4.276 23908574 PMC3730070

[16] Kiziltunc, PB, Atilla, H, Çalis, F and Alay, C. 2016 Comparison of surgical success for infantile esotropia and strabismus associated with neurological impairment. Strabismus, 24: 97–100. DOI: 10.1080/09273972.2016.1210173 27532638

[17] Liu, G and Ranka, P. 2014 Strabismus surgery for children with developmental delay. Current Opinion in Ophthalmology, 25: 417–423. DOI: 10.1097/ICU.0000000000000086 25050756

[18] Ljubic, A, Trajkovski, V and Stankovic, B. 2011 Strabismus, refractive errors and nystagmus in children and young adults with Down syndrome. Ophthalmic Genetics, 32: 204–211. DOI: 10.3109/13816810.2011.592175 21728809

[19] Ljubic, A, Trajkovski, V, Tesic, M, Tojtovska, B and Stankovic, B. 2015 Ophthalmic manifestations in children and young adults with Down syndrome and congenital heart defects. Ophthalmic Epidemiology, 22: 123–129. DOI: 10.3109/09286586.2015.1017652 25777312

[20] Louwagie, CR, Greenberg, AE, Mohney, BG and Diehl, NN. 2009 Is the incidence of infantile esotropia declining? A population-based study from Olmsted County, Minnesota, 1965 to 1994. Archives of Ophthalmology, 127: 200–203. DOI: 10.1001/archophthalmol.2008.568 19204240 PMC2762940

[21] Majnemer, A and Shevell, MI. 1995 Diagnostic yield of the neurologic assessment of the developmentally delayed child. The Journal of Pediatrics, 127: 193–199. DOI: 10.1016/S0022-3476(95)70294-6 7543566

[22] Mithyantha, R, Kneen, R, McCann, E and Gladstone, M. 2017 Current evidence-based recommendations on investigating children with global developmental delay. Archives of Disease in Childhood, 102: 1071 DOI: 10.1136/archdischild-2016-311271 29054862 PMC5738593

[23] Motley, WW, Melson, AT, Gray, ME and Salisbury, SR. 2012 Outcomes of strabismus surgery for esotropia in children with Down syndrome compared with matched controls United States: Slack Incorporated DOI: 10.3928/01913913-20120207-04 22329551

[24] Nielsen, LS, Skov, L and Jensen, H. 2007 Visual dysfunctions and ocular disorders in children with developmental delay: I. prevalence, diagnoses and aetiology of visual impairment. Acta Ophthalmologica Scandinavica, 85: 149–156. DOI: 10.1111/j.1600-0420.2006.00867.x 17263780

[25] Park, YC, Chun, BY and Kwon, JY. 2009 Comparison of the stability of postoperative alignment in sensory exotropia: adjustable versus non-adjustable surgery. Korean Journal Of Ophthalmology, 23: 277–280. DOI: 10.3341/kjo.2009.23.4.277 20046688 PMC2789952

[26] Perez, CI, Zuazo, F, Zanolli, MT, Guerra, JP, Acuña, O and Iturriaga, H. 2013 Major article: Esotropia surgery in children with Down syndrome. Journal of AAPOS, 17: 477–479. DOI: 10.1016/j.jaapos.2013.06.007 24160966

[27] Pickering, JD, Simon, JW, Lininger, LL, Melsopp, KB and Pinto, GL. 1994 Exaggerated effect of bilateral medial rectus recession in developmentally delayed children. Journal of Pediatric Ophthalmology and Strabismus, 31: 374–377. 7536239 10.3928/0191-3913-19941101-06

[28] Pickering, JD, Simon, JW, Ratliff, CD, Melsopp, KB and Lininger, LL. 1995 Alignment success following medial rectus recessions in normal and delayed children. Journal of Pediatric Ophthalmology and Strabismus, 32: 225–227. DOI: 10.3928/0191-3913-19950701-05 7494157

[29] Pukrushpan, P. 2009 Drift of ocular alignment following strabismus surgery. Part 1: Using fixed scleral sutures. British Journal of Ophthalmology, 93: 439–442. DOI: 10.1136/bjo.2007.136390 18617540

[30] Robaei, D, Wand, JJ, Tan, M, Rose, KA, Kifley, A and Mitchell, P. 2006 Patterns of eyecare utilization by young Australian children: Findings from a population-based study. Ophthalmic Epidemiology, 13: 153–158. DOI: 10.1080/09286580600630187 16854768

[31] Ruttum, MS, Kivlin, JD and Hong, P. 2004 Outcome of surgery for esotropia in children with Down syndrome. American Orthoptic Journal, 54: 98–101. DOI: 10.3368/aoj.54.1.98 21149092

[32] Shevell, M, Majnemer, A, Ashwal, S, Flint, J, Hirtz, D, Noetzel, M, et al. 2003 Practice parameter: Evaluation of the child with global developmental delay: Report of the quality standards subcommittee of the American academy of neurology and the practice committee of the child neurology society. Neurology, 60: 367–380. DOI: 10.1212/01.WNL.0000031431.81555.16 12578916

[33] Simonsz, HJ and Kolling, GH. 2011 Best age for surgery for infantile esotropia. European Journal of Paediatric Neurology, 15: 205–208. DOI: 10.1016/j.ejpn.2011.03.004 21511504

[34] Swaminathan, M, Shah, SV, Mittal, S and Gunasekaran, A. 2014 Results of bilateral medial rectus recession for comitant esotropia in patients with developmental delay. Strabismus, 22: 138–142. DOI: 10.3109/09273972.2014.907814 24798741

[35] Von Noorden, GK. 1988 A reassessment of infantile esotropia XLIV Edward Jackson memorial lecture. American Journal of Ophthalmology, 105: 1–10. DOI: 10.1016/0002-9394(88)90113-4 3337186

[36] Wang, L and Wand, X. 2012 Comparison between graded unilateral and bilateral medial rectus recession for esotropia. British Journal of Ophthalmology, 96: 540–543. DOI: 10.1136/bjophthalmol-2011-300932 22096137

[37] Yahalom, C, Mechoulam, H, Cohen, E and Anteby, I. 2010 Major article: Strabismus surgery outcome among children and young adults with Down syndrome. Journal of AAPOS, 14: 117–119. DOI: 10.1016/j.jaapos.2010.01.009 20451852

[38] Yurdakul, NS, Ugurlu, S and Maden, A. 2006 Strabismus in Down syndrome. Journal Of Pediatric Ophthalmology And Strabismus, 43: 27–30. DOI: 10.3928/01913913-20060101-03 16491722

[39] Zahidi, AAA, Vinuela-Navarro, V and Woodhouse, JM. 2018 Different visual development: norms for visual acuity in children with Down syndrome. Clinical and Experimental Optometry, 101: 535–540. DOI: 10.1111/cxo.12684 29601092

[40] Zehavi-Dorin, T, Ben-Zion, I, Mezer, E and Wyganski-Jaffe, T. 2016 Long-term results of bilateral medial rectus muscle recession in children with developmental delay. Strabismus (09273972), 24: 7–11. DOI: 10.3109/09273972.2015.1130064 26954620

